# TORC1 coordinates the conversion of Sic1 from a target to an inhibitor of cyclin-CDK-Cks1

**DOI:** 10.1038/celldisc.2017.12

**Published:** 2017-05-02

**Authors:** Marta Moreno-Torres, Malika Jaquenoud, Marie-Pierre Péli-Gulli, Raffaele Nicastro, Claudio De Virgilio

**Affiliations:** 1 Department of Biology, University of Fribourg, Fribourg, Switzerland

**Keywords:** target of rapamycin complex 1 (TORC1), cyclin-dependent protein kinase (CDK), CDK inhibitor (CDKI), G_1_ cell cycle arrest, greatwall kinase pathway, Cks1, Sic1, Rim15

## Abstract

Eukaryotic cell cycle progression through G_1_–S is driven by hormonal and growth-related signals that are transmitted by the target of rapamycin complex 1 (TORC1) pathway. In yeast, inactivation of TORC1 restricts G_1_–S transition due to the rapid clearance of G_1_ cyclins (Cln) and the stabilization of the B-type cyclin (Clb) cyclin-dependent kinase (CDK) inhibitor Sic1. The latter mechanism remains mysterious but requires the phosphorylation of Sic1-Thr^173^ by Mpk1 and inactivation of the Sic1-pThr^173^-targeting phosphatase (PP2A^Cdc55^) through greatwall kinase-activated endosulfines. Here we show that the Sic1-pThr^173^ residue serves as a specific docking site for the CDK phospho-acceptor subunit Cks1 that sequesters, together with a C-terminal Clb5-binding motif in Sic1, Clb5-CDK-Cks1 complexes, thereby preventing them from flagging Sic1 for ubiquitin-dependent proteolysis. Interestingly, this functional switch of Sic1 from a target to an inhibitor of cyclin-CDK-Cks1 also operates in proliferating cells and is coordinated by the greatwall kinase, which responds to both Cln-CDK-dependent cell-cycle and TORC1-mediated nutritional cues.

## Introduction

Eukaryotic cell proliferation requires proper coordination of growth with key cell cycle events such as the G_1_–S cell cycle transition, which is also coined START in yeast [1]. Initiation of and commitment to a new round of cell division at START depends on the G_1_ cyclin-dependent protein kinase (Cln-CDK; Cln1/2-Cdc28) that phosphorylates critical target proteins [2]. One of these is the B-type cyclin (Clb)-CDK inhibitor (CDKI) Sic1 [3], which, following its CDK-dependent phosphorylation at multiple residues [4], associates with the F-box protein Cdc4 of the SCF^Cdc4^ ubiquitin ligase complex that directs it for ubiquitin-dependent proteolysis (for reviews, see Deshaies *et al.* [5] and Barberis [6]). The phosphorylation of Sic1 in late G_1_ follows a temporal and spatial order that involves an initial Cln-CDK-dependent phosphorylation of Thr^5^ and Thr^33^ within its N-terminal region [7]. This priming event creates docking sites for the phospho-acceptor subunit Cks1 of Cln-CDK-Cks1 and emerging Clb5/6-CDK-Cks1 complexes, which subsequently phosphorylate critical pairs of neighboring residues (Thr^45^/Thr^48^ and Ser^76^/Ser^80^; also coined phosphodegrons [8]) that engage Cdc4 to mark Sic1 for destruction [7, 9, 10]. Both the synergistic effect between Cln- and Clb-CDKs in semi-processive, multisite-phosphorylation of Sic1 and the positive feedback loop involving liberated Clb5/6-CDK-Cks1 complexes render the destruction of Sic1 switch-like and are therefore key for launching the G_1_–S cell cycle transition in yeast [11].

Consistent with the important role of Sic1 degradation for G_1_–S transition, this process is tightly coupled to growth- and nutrient-related signals that are mediated by TORC1, the central controller of growth in eukaryotes [12, 13]. Accordingly, TORC1 stimulates the formation of Cln-CDK complexes that prime Sic1 for multisite-phosphorylation by activating the transcription and translation of G_1_ cyclins (Cln1–3) [14, 15]. In addition, TORC1 also antagonizes the phosphorylation of a specific residue (Thr^173^) within the C-terminal part of Sic1 that critically contributes to its stabilization [16]. TORC1 controls the phosphorylation status of this residue via two circuits, of which one involves the indirect inhibition of the mitogen-activated protein kinase Slt2/Mpk1 that phosphorylates Thr^173^ in Sic1 [17]. In parallel, TORC1 warrants effective dephosphorylation of pThr^173^ in Sic1 by inhibiting a signaling branch, which restrains the activity of the Sic1-pThr^173^-targeting PP2A^Cdc55^ protein phosphatase [17]. The core of this highly conserved signaling branch consists of the greatwall kinase (Gwl) Rim15, which phosphorylates a residue within the endosulfines Igo1/2 to convert them to potent inhibitors of PP2A^Cdc55^ (or B55-PP2A in higher eukaryotes) [18–20]. TORC1 contributes to the silencing of Rim15 by promoting indirectly the phosphorylation of two residues (that is, Ser^1061^ and Thr^1075^), which cooperatively mediate tandem 14-3-3 protein binding to sequestrate inactive Rim15 in the cytoplasm [21–24]. Downregulation of TORC1 therefore results in the release of Rim15 from its cytoplasmic anchor, enabling it to accumulate in the nucleus to activate the PP2A^Cdc55^ inhibitory Igo1/2 proteins. Combined with the coordinated activation of Mpk1, the latter process ensures the phosphorylation of Sic1 at Thr^173^ under conditions that inhibit TORC1 such as nutrient limitation or rapamycin treatment. How this particular phosphorylation event stabilizes Sic1 remains currently mysterious.

Here we show that the phosphorylation of Thr^173^ in Sic1 creates a docking site for the phospho-acceptor subunit Cks1 that tethers Clb5-CDK-Cks1 complexes away from the respective N-terminal docking sites on Sic1 where they could otherwise favor multi-phosphorylation and degradation of Sic1. The spatial sequestration of the pThr^173^-associated Clb5-CDK-Cks1 complexes also promotes their inhibition via the additional association of Clb5 with the RXL motif within the CDK-inhibitory C-terminus (RXL^CDKI^) of Sic1. Thus TORC1 inactivation (and consequently Mpk1 and Gwl pathway activation) triggers the phosphorylation of Thr^173^ in Sic1, thereby converting Sic1 from a target to a potent inhibitor of Clb5-CDK-Cks1 complexes. Interestingly, this switch of Sic1 also occurs in proliferating cells in G_1_ and is rapidly reversed at START as a result of the increase of Cln-CDK, which, cooperatively with TORC1, inhibits Rim15 by stimulating its confinement to the cytoplasm. Thus, our data pinpoint the Gwl pathway as a central node that interprets both growth and cell cycle cues to coordinate G_1_–S transition.

## Results and Discussion

### Phosphorylation of Sic1-Thr^173^ is essential for Sic1 to escape CDK-mediated, N-terminal multi-phosphorylation upon TORC1 inactivation

**Figure 1:**
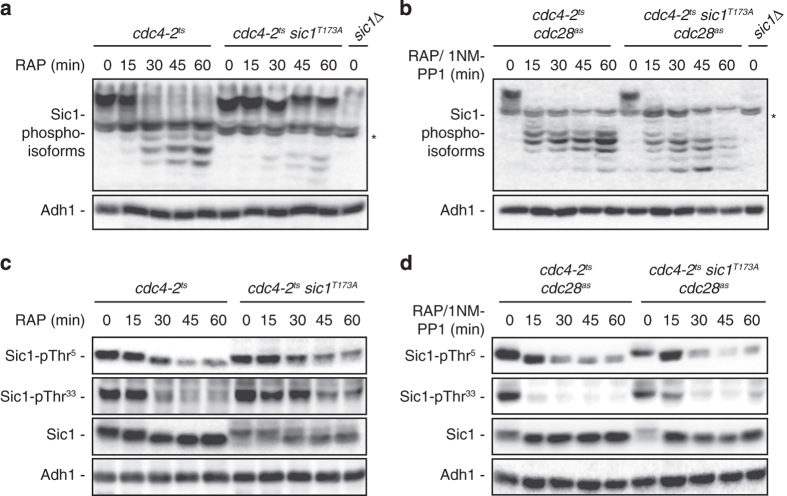
Sic1-Thr^173^ mediates protection from cyclin-dependent kinase (CDK)-dependent multi-phosphorylation upon target of rapamycin complex 1 (TORC1) inactivation. (**a**) Sic1^T173A^ is hyperphosphorylated in rapamycin-treated *cdc4-2*^*ts*^ cells. *cdc4-2*^*ts*^ and *cdc4-2*^*ts*^ *sic1*^*T173A*^ mutant cells were incubated for 3 h at 37 °C, then treated (RAP, 15–60 min), or not (RAP, 0 min), with rapamycin. Sic1 and Sic1^T173A^ were analyzed using polyclonal anti-Sic1 antibodies and phos-tag phosphate affinity gel electrophoresis. The asterisk denotes unspecific bands that were also present in *sic1*Δ control cells. (**b**) Inactivation of Cdc28 prevents hyperphosphorylation of Sic1^T173A^ in rapamycin-treated *cdc4-2*^*ts*^ cells. Sic1 and Sic1^T173A^ were analyzed as in **a** using *cdc4-2*^*ts*^ *cdc28*^*as*^ and *cdc4-2*^*ts*^ *sic1*^*T173A*^ *cdc28*^*as*^ strains. 1NM-PP was added simultaneously with rapamycin. (**c**) Sic1^T173A^ is hyperphosphorylated at Thr^5^ and Thr^33^ in rapamycin-treated *cdc4-2*^*ts*^ cells. Strains and conditions were as in **a**. Sic1-pThr^5^ and Sic1-pThr^33^ levels were determined by immunoblot analysis using phospho-specific anti-Sic1-pThr^5^ and anti-Sic1-pThr^33^ antibodies, respectively (see Supplementary Figure S1). Sic1 was probed with polyclonal anti-Sic1 antibodies. Notably, hyperphosphorylation of Sic1^T173A^ causes it to migrate over a larger region within standard 9% sodium dodecyl sulfate (SDS) gels, which is why the respective protein bands appear less sharp and intense when probed with polyclonal anti-Sic1 antibodies (third panel from top; lanes 6–10). (**d**) Inactivation of Cdc28 prevents hyperphosphorylation of Thr^5^ and Thr^33^ in Sic1^T173A^ in rapamycin-treated *cdc4-2*^*ts*^ cells. Strains and conditions were as in **b**. Sic1-pThr^5^, Sic1-pThr^33^ and Sic1 were determined as in **c**. As in **c**, hyperphophorylated Sic1^T173^ at time point 0 (third panel from the top; lane 6) migrates over a larger region within the SDS gel. Inactivation of Cdc28^as^ (lanes 7–10), however, results in a rapid dephosphorylation of Sic1^T173A^, which is why the respective protein bands collapse into a sharper and intense band under these conditions. In all panels, Adh1 levels served as loading control.

Previous studies suggested that the phosphorylation of Thr^173^ in Sic1 may compromise its capacity to interact with components of the SCF^Cdc4^ complex [16, 25]. As the respective interaction is mainly mediated by a set of defined N-terminal phosphodegron sites [4, 26], we considered the possibility that pThr^173^ may, rather than directly interfering with SCF^Cdc4^ binding, indirectly affect the phosphorylation status of the N-terminal residues in Sic1. To address this possibility, we first compared on phos-tag gels the phosphorylation levels of Sic1 and Sic1^T173A^ from rapamycin-treated *cdc4-2*^*ts*^ strains, which were preincubated at the restrictive temperature (that is, 37 °C) to inactivate Cdc4 and consequently block SCF^Cdc4^-mediated degradation of the Sic1 variants. Under these conditions, the slowly migrating, hyperphosphorylated wild-type (WT) Sic1, but not the Sic1^T173A^ variant, rapidly collapsed into several fast-migrating, hypophosphorylated isoforms (Figure 1a). Inhibition of Cdc28 during the rapamycin treatment (using the ATP analog 1NM-PP1 and strains carrying the analog-sensitive Cdc28^as^ allele) prevented the observed sustained hyperphosphorylation of Sic1^T173A^, indicating that Cln- and/or Clb-CDK complexes are not properly inhibited in Sic1^T173A^-expressing cells (Figure 1b). This was further supported by our finding that both the Sic1-Thr^5^ and Sic1-Thr^33^ residues, which are preferred Cln2-CDK and Clb5-CDK targets in Sic1, respectively [7], were swiftly dephosphorylated in rapamycin-treated *cdc4-2*^*ts*^ cells, while this effect was significantly delayed in *cdc4-2*^*ts*^ *sic1*^*T173A*^ cells (Figure 1c). Using strains that carry the Cdc28^as^ allele and 1NM-PP1 to inactivate Cdc28^as^ during the rapamycin treatment as above, we confirmed that the delayed response in pThr^5^ and pThr^33^ dephosphorylation in rapamycin-treated, Sic1^T173A^-expressing cells was entirely dependent on Cdc28 (Figure 1d). Collectively, these results indicate that the instability of the Sic1^T173A^ allele results primarily from its sustained phosphorylation by Cln- and/or Clb-CDK.

### The Sic1^T173A^ allele exhibits normal specificity and intrinsic CDKI activity in vitro

**Figure 2:**
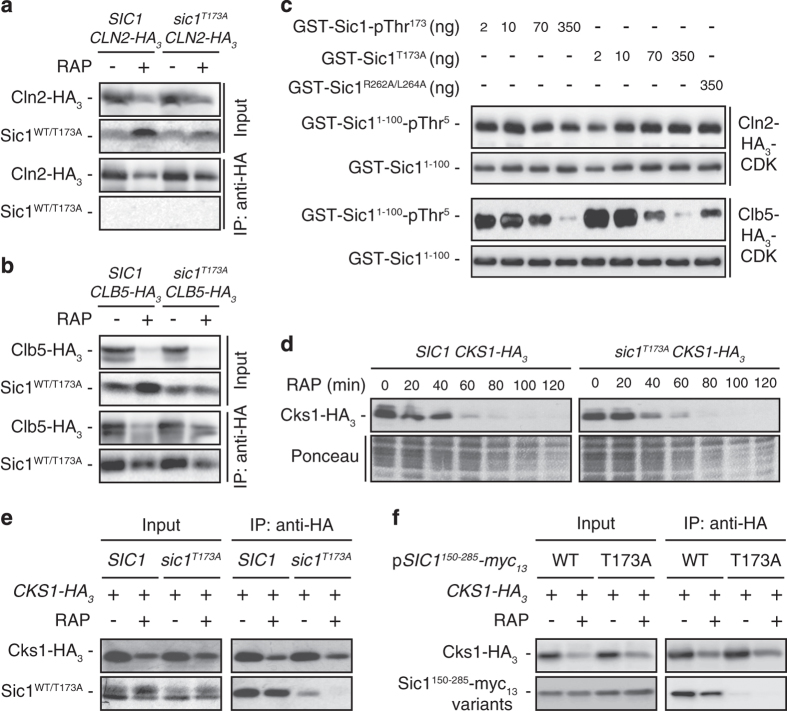
Sic1-pThr^173^ acts as a docking site for Cks1. (**a**, **b**) Sic1 and Sic1^T173A^ bind Clb5 but not Cln2. HA_3_-tagged Cln2 (**a**) or Clb5 (**b**) were immunoprecipitated (IPed) from extracts of exponentially growing wild-type (WT; *SIC1*) and *sic1*^*T173A*^ cells that were treated (RAP, +), or not (RAP, −), for 1 h with rapamycin. Cell lysates (Input) and anti-HA immunoprecipitates (IP: anti-HA) were analyzed by immunoblotting with anti-HA and anti-Sic1 antibodies. See also Supplementary Figure S3 for additional control IPs. (**c**) Sic1 and Sic1^T173A^ inhibit Clb5-CDK, but not Cln2-CDK, *in vitro*. Cln2-HA_3_-CDK and Clb5-HA_3_-CDK immunocomplexes from exponentially growing yeast cells were used for *in vitro* kinase assays in which the bacterially purified, N-terminal part of Sic1 (GST-Sic1^1-100^) served as substrate. Bacterially purified GST-Sic1, GST-Sic1^T173A^ or GST-Sic1^R262A/L264A^ (that were subjected to *in vitro* phosphorylation by Mpk1 before use) were titrated into the reactions at the indicated concentrations to assess their cyclin-dependent kinase (CDK) inhibitory activities. CDK activity was assessed by probing the levels of pThr^5^ in GST-Sic1^1-100^ using anti-Sic1-pThr^5^ antibodies. GST-Sic1^1-100^ levels were verified by immunoblot analysis using anti-GST antibodies. (**d**) Rapamycin-treated Sic1^T173A^-expressing cells are not defective in the clearance of Cks1. Exponentially growing WT and *sic1*^*T173A*^ cells expressing genomically tagged Cks1-HA_3_ were treated (RAP, 20–120 min), or not (RAP, 0 min), with rapamycin. Cks1-HA_3_ levels were determined by immunoblot analysis (using monoclonal anti-HA antibodies). Ponceau staining served as loading control. (**e**, **f**) Cks1 binds Sic1, but not Sic1^T173A^, preferably in rapamycin-treated cells. Genomically HA_3_-tagged Cks1 was IPed from extracts of exponentially growing (RAP, −) or rapamycin-treated (1 h; RAP, +) WT or *sic1*^*T173A*^ cells (**e**) or from respective WT cells that expressed plasmid-encoded myc_13_-tagged Sic1 fragments (Sic1^150-285^-myc_13_) carrying (T173A), or not (WT), the Thr^173^-to-Ala mutation (**f**). Cell lysates (Input) and anti-HA immunoprecipitates (IP: anti-HA) were analyzed by immunoblotting with anti-HA and anti-Sic1 (**e**) or anti-myc (**f**) antibodies.

To elucidate why rapamycin-treated *sic1*^*T173A*^ cells exhibit enhanced Cln- and/or Clb-CDK activity, we next analyzed whether the mutation of Thr^173^ in Sic1 indirectly affected the levels of G_1_- (Cln1/2) or S-phase (Clb5) cyclins. As shown in Supplementary Figure S2, Cln1, Cln2 and Clb5 were all eliminated with similar kinetics in both rapamycin-treated *cdc4-2*^*ts*^ and *cdc4-2*^*ts*^ *sic1*^*T173A*^ cells. Expression of the Sic1^T173A^ allele does therefore not alter the dynamic regulation of G_1_/S cyclin levels in rapamycin-treated cells. Because Sic1^T173A^-expressing cells exhibited sustained phosphorylation of a preferred Cln-CDK target (that is, Thr^5^ in Sic1; see above), we next speculated that the phosphorylation of Thr^173^ in Sic1 may expand its inhibitory specificity in rapamycin-treated cells to include Cln-CDKs. However, this cannot be the case as Cln2 did not bind Sic1 or Sic1^T173A^ under any conditions tested (Figure 2a), while Clb5 interacted readily with both forms in exponentially growing and rapamycin-treated cells (Figure 2b). Thus neither the phosphorylation (in rapamycin-treated WT cells) nor the mutation of Thr^173^ appeared to modify the specificity of Sic1 for binding Clb5-CDK *in vivo*. We therefore addressed yet another possibility, namely that the Thr^173^ residue may be important for the capacity of Sic1 to function as CDKI. To this end, we used bacterially expressed Sic1 and Sic1^T173A^ that were exposed to the Thr^173^-targeting Mpk1 kinase prior to their use in CDKI assays. These revealed that both Sic1-pThr^173^ and Sic1^T173A^ inhibited Clb5-Cdc28, but not Cln2-Cdc28, equally well in a concentration-dependent manner *in vitro* (Figure 2c). Unlike the Sic1^R262A/L264A^ allele, which carries specific mutations in the RXL^CDKI^ motif that likely tethers the hydrophobic patch (hp) docking site in Clb5 (Figure 2c; see also below) [27–29], the recombinant Sic1^T173A^ mutant is therefore not intrinsically deficient in Clb5-CDK inhibition when assayed *in vitro*.

### Sic1-pThr^173^ acts as a docking site for the CDK phospho-adaptor subunit Cks1

An alternative model we entertained to explain the higher Clb/Cln-CDK activity in Sic1^T173A^ expressing cells is that the phosphorylation of Thr^173^ in Sic1 indirectly contributes to CDK inhibition by sequestering a protein that otherwise favors the capacity of cells to stimulate CDKs. To identify such a protein(s), we purified Sic1 and Sic1^T173A^ from rapamycin-treated cells and analyzed the coprecipitating proteins by mass spectrometry. Remarkably, when applying stringent criteria, this experiment pinpointed Cks1 as the only protein that interacted specifically and more robustly with Sic1 when compared with Sic1^T173A^. To exclude that the presumably lower affinity between Cks1 and Sic1^T173A^ is simply due to lower Cks1 levels in *sic1*^*T173A*^ strains, we compared the Cks1 expression levels in WT and *sic1*^*T173A*^ cells. Cks1 levels were equally high in exponentially growing cells and rapidly dropped with similar kinetics in rapamycin-treated cells of both strains (Figure 2d). Directed control co-immunoprecipitation (co-IP) analyses, in which we pulled down HA_3_-tagged Cks1 and assessed the levels of associated Sic1 or Sic1^T173A^, also confirmed our finding that Cks1 associates with Sic1, but not with Sic1^T173A^, in rapamycin-treated cells (Figure 2e). These results indicate that the CDK phospho-adaptor subunit Cks1 specifically docks to pThr^173^ within Sic1. However, the interpretation of these experiments may be subjected to the following caveats, namely (i) that the lysates from rapamycin-treated *sic1*^*T173A*^ cells contained much lower amounts of the instable Sic1^T173A^ than the ones of WT cells containing the stable Sic1 protein, and (ii) that our co-IP experiments may, to some extent, probe the association of Cks1 with the N-terminal Cks1-docking sites in Sic1/Sic1^T173A^. To address both issues at once, we carried out additional Cks1 co-IP experiments using myc_13_-tagged fragments of Sic1 (Sic1^150-285^), which lacked the N-terminal phosphodegron and Cks1-docking sites that drive Sic1 degradation. As expected, these Sic1 fragments, whether they contained or not the Thr^173^-to-Ala mutation, were equally stable under all conditions tested. The respective co-IP experiments with Cks1 confirmed the ones in which full-length variants of Sic1 were expressed (Figure 2e and f). Together with the fact that the pThr^173^ and its neighboring residues (Pro-Gly-Thr^173^-Pro) perfectly match the Cks1-binding consensus motif (F/I/L/P/V/W/Y)XTP [10], our results therefore assign pThr^173^ a role as *bona fide* Cks1-docking site.

### The C-terminal RXL motif and the pThr^173^ residue coordinately bind Clb5-CDK-Cks1

**Figure 3:**
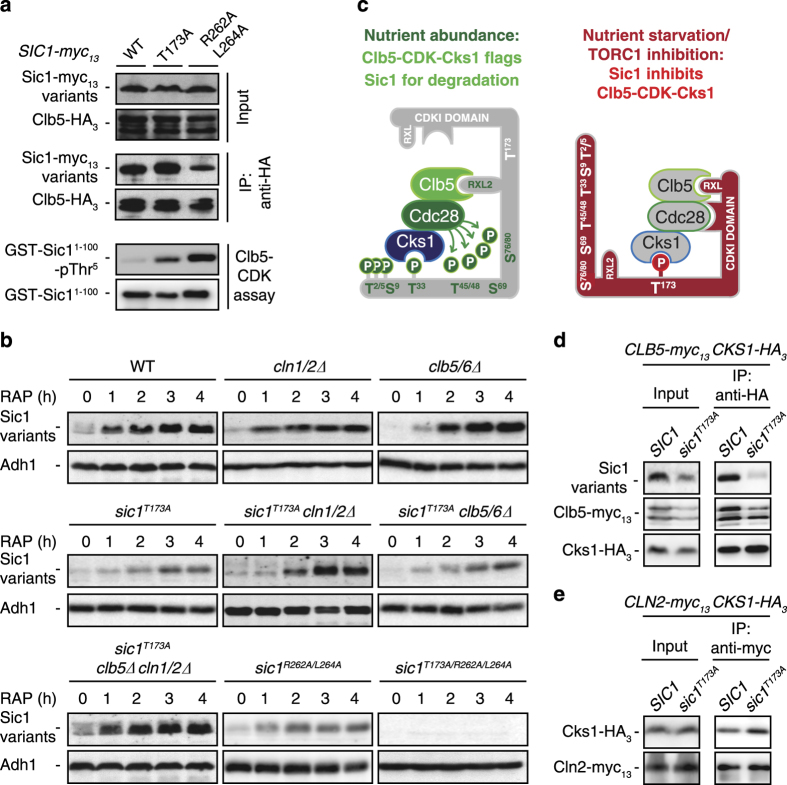
The C-terminal RXL-motif and pThr^173^ coordinately bind Clb5-CDK-Cks1. (**a**) Clb5-CDK immune complexes from rapamycin-treated *sic1*^*T173A*^ and *sic1*^*R262A/L264A*^ cells, but not the ones from respective wild-type (WT) cells, exhibit cyclin-dependent kinase (CDK) activity. Plasmid-expressed Clb5-HA_3_ was IPed from extracts of rapamycin-treated (1 h) cells that expressed genomically tagged Sic1-myc_13_-, Sic1^T173A^-myc_13_- or Sic1^R262A/L264A^-myc_13_. Cell lysates (Input) and anti-HA immunoprecipitates (IP: anti-HA) were analyzed by immunoblotting with anti-HA and anti-myc antibodies. Clb5-CDK immune complexes were probed for CDK activity as in Figure 2c. (**b**) Combined loss of Cln1/2 and Clb5 suppresses the instability of Sic1^T173A^ in rapamycin-treated cells. The levels of Sic1 variants were determined in exponentially growing (RAP, 0 h) and rapamycin-treated (1–4 h) strains with the indicated genotypes using polyclonal anti-Sic1 antibodies. Adh1 levels served as loading controls. (**c**) Model for the target of rapamycin complex 1 (TORC1)-/nutrient-regulated switch of Sic1 from a target to an inhibitor of Clb5-CDK-Cks1. In cells growing on rich medium, Cks1-CDK-Clb5 is found to dock onto Sic1 via Cks1 binding to pThr^5^ and pThr^33^ (only the latter binding is shown) and via Clb5 binding to RXL motifs (only RXL2 of four RXL motifs is shown). This allows phosphorylation of the neighboring phosphodegron clusters at Thr^45/48^ and Ser^69/76/80^ [7, 9, 10] and subsequent degradation of Sic1. Upon TORC1 inactivation or nutrient starvation, a distinct docking configuration of Cks1-CDK-Clb5 onto Sic1 happens, involving binding of Cks1 to Thr^173^ and of Clb5 to a C-terminal RXL motif within the CDKI domain. This precludes further phosphorylation of Sic1 by Cks1-CDK-Clb5 and favors its stabilization. For further details, see text. (**d**) Cks1-Clb5 complexes are released from CDKI-binding in rapamycin-treated *sic1*^*T173A*^ cells. Genomically tagged Cks1-HA_3_ was IPed from extracts of rapamycin-treated (30 min) WT (*SIC1*) or *sic1*^*T173A*^ cells that co-expressed genomically tagged Clb5-myc_13_. Cell lysates (Input) and anti-HA immunoprecipitates (IP: anti-HA) were analyzed by immunoblotting with anti-myc, anti-HA, and anti-Sic1 antibodies. (**e**) Rapamycin-treated *sic1*^*T173A*^ cells exhibit higher levels of Cks1-Cln2 complexes. Genomically tagged Cln2-myc_13_ was IPed from extracts of rapamycin-treated (30 min) WT (*SIC1*) or *sic1*^*T173A*^ cells that co-expressed genomically-tagged Cks1-HA_3_. Cell lysates (Input) and anti-HA immunoprecipitates (IP: anti-HA) were analyzed by immunoblotting with anti-myc and anti-HA antibodies.

Our co-IP studies presented in Figure 2e and f indicated that Cks1 also associates with Sic1, in a Thr^173^-dependent manner, in exponentially growing cells. This is not surprising, as low levels of Sic1-pThr^173^ molecules can also be detected in these cells [17] (see also below). Importantly, however, rapamycin treatment strongly increased the relative amount of Sic1 that was precipitated with Cks1 (Figure 2e and f), suggesting that Cks1 molecules, if not degraded, get rapidly trapped by Sic1-pThr^173^ in rapamycin-treated cells. This mechanism likely contributes to the swift clearance of free Cks1 molecules and may potentially contribute to a more robust inhibition of Clb5-CDK-Cks1 complexes due to their insertion between the Clb5-tethering RXL^CDKI^ domain and the pThr^173^ Cks1-docking site in Sic1. Several additional experiments support this model. For instance, mutation of the RXL^CDKI^ motif in Sic1 strongly weakened the association between Clb5 and this Sic1 mutant protein. This resulted in the release of Clb5-CDK from Sic1 inhibition, as Clb5-CDK immune complexes isolated from rapamycin-treated Sic1^R262A/L264A^-expressing cells (unlike the ones from WT cells) exhibited largely unrestrained Clb5-CDK activity *in vitro* (Figure 3a). In addition, even though Clb5 pulled down Sic1 and Sic1^T173A^ with equal efficiency from extracts of rapamycin-treated cells (see also Figure 2b), only Clb5-CDK-Sic1^T173A^ immune complexes exhibited measurable levels of unrestrained Clb5-CDK activity (Figure 3a). These data show that the capacity of Sic1 to dock Cks1 on pThr^173^ grants more stringent inhibition of Clb5-CDK *in vivo*. This also explains why recombinant Sic1 and Sic1^T173A^ were found to be equally effective CDKIs when assayed *in vitro* in the absence of Cks1 (Figure 2c). Lastly, as expected if both Sic1^R262A/L264A^ and Sic1^T173A^ were defective in separate mechanisms to adequately inhibit of Clb5-CDK-Cks1 complexes *in vivo* (and hence were prone themselves to continued flagging for degradation by the latter complexes), both Sic1 variants were unstable in an additive manner when assayed in rapamycin-treated cells (Figure 3b). Thus the robust inhibition of Clb5-CDK-Cks1 complexes *in vivo* requires the coordinate binding of Clb5 to RXL^CDKI^ and Cks1 to pThr^173^ within Sic1 (Figure 3c).

Our results imply that the sequestration of Cks1 within Sic1-associated Clb5-CDK-Cks1 complexes is part of a program that prevents Cks1 from directing free Cln/Clb-CDKs toward substrates (including Sic1 itself) when TORC1 is low. Our co-IP analyses using extracts from rapamycin-treated cells endorsed this hypothesis, as Cks1 interacted well with Clb5 and Sic1 in WT cells, while Cks1 only bound Clb5 but not Sic1^T173A^ in *sic1*^*T173A*^ cells (Figure 3d). Under the same conditions, Cks1 also increasingly bound Cln2 in *sic1*^*T173A*^ cells when compared with WT cells (Figure 3e). Finally, our genetic experiments revealed that only the combined loss of Clb5 and Cln1/2, but not their individual loss, fully suppressed the instability of the Sic1^T173A^ allele in rapamycin-treated cells. The B-type cyclin-CDKI Sic1 therefore has a hitherto undescribed role in limiting the activity of Cln-CDKs by sequestering their regulatory adaptor Cks1 upon TORC1 inactivation.

### The Gwl Rim15 integrates signals from both TORC1 and Cln-CDK to control Sic1

**Figure 4:**
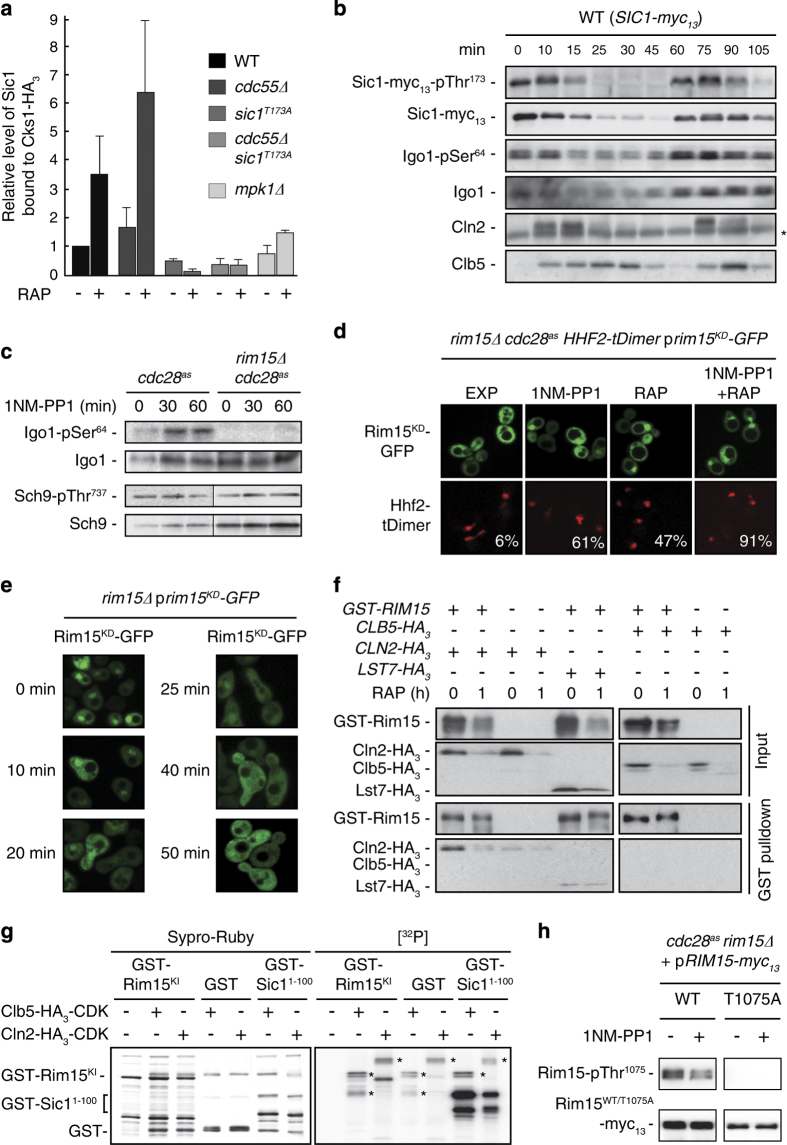
The Gwl Rim15 integrates target of rapamycin complex 1 (TORC1) and Cln-CDK signals to control the G_1_–S transition. (**a**) The Gwl pathway controls the association between Cks1 and Sic1. Genomically HA_3_-tagged Cks1 was IPed from extracts of exponentially growing cells (genotypes indicated) that were treated (RAP, +), or not (RAP, −), for 2 h with rapamycin. Bars denote the relative amount of Sic1 that co-precipitated with Cks1 (see also Figure 2e for details). The respective ratios were all normalized to the value in exponentially growing wild-type (WT) cells (*n*=3; ±s.d.). (**b**) Rim15 activity is cell cycle regulated. For synchronization, exponentially growing WT cells expressing genomically tagged Sic1-myc_13_ were treated for 2 h with α-factor (5 μg ml^−1^). Following α-factor release, samples were collected at the indicated time points. Sic1-myc_13_-pThr^173^, Sic1-myc_13_, Igo1-pSer^64^, Igo1, Cln2 and Clb5 levels were detected by immunoblot analysis using anti-pThr^173^, anti-myc, anti-Igo1-pSer^64^, anti-Igo1, anti-Cln2 and anti-Clb5 antibodies, respectively. Notably, we have used our anti-Sic1-pThr^173^ and Igo1-pSer^64^ antibodies in previous studies in which we have also shown that both antibodies recognize the respective phospho-residues in Sic1 (pThr^173^) and Igo1 (Ser^64^) with high specificity [17, 31]. The asterisk denotes an unspecific band. (**c**) Cdc28 antagonizes Igo1-Ser^64^ phosphorylation. Exponentially growing (0 min) *cdc28*^*as*^ and *rim15*Δ *cdc28*^*as*^ cells were treated for the times indicated with 1NM-PP1 (0.5 μM) to inactivate Cdc28^as^. The levels of both Igo1 and the phosphorylation of the Rim15-target residue Ser^64^ in Igo1 were determined by immunoblot analysis using anti-Igo1 and phospho-specific anti-Igo1-pSer^64^ antibodies. To assay TORC1 activity, the levels of both Sch9 and the phosphorylation of the TORC1-target residue Thr^737^ in Sch9 were determined by immunoblot analysis using anti Sch9 and phospho-specific anti-Sch9-pThr^737^ antibodies. (**d**) *rim15*Δ *cdc28*^*as*^ cells expressing plasmid-encoded kinase-inactive GFP-Rim15^KD^ and the genomically tagged nuclear marker Hhf2-tDimer were grown exponentially. They were left untreated (EXP), or treated for 1 h with 1NM-PP1, rapamycin (RAP) or 1NM-PP1 combined with rapamycin. The subcellular distribution of green fluorescent protein (GFP)-Rim15^KD^ and Hhf2-tDimer was analyzed by fluorescence microscopy. The numbers indicate the percentage of cells (*n*>100) with clear nuclear accumulation of the GFP-fusion protein. (**e**) Exponentially growing *rim15*Δ cells expressing plasmid-encoded GFP-Rim15^KD^ were synchronized by α-factor treatment as in (**b**) and analyzed by fluorescence microscopy at the times indicated following α-factor release. (**f**) Cln2-CDK but not Clb5-CDK complexes interact with Rim15 in exponentially growing cells. Plasmid-encoded GST-Rim15 was pulled down from extracts of untreated (0 h) and rapamycin-treated (RAP; 1 h) *rim15*Δ cells that co-expressed Clb5-HA_3_, Cln2-HA_3_ or Lst7-HA_3_. Cells carrying an empty vector were used as control. Cell lysates (Input) and GST pulldown fractions were analyzed by immunoblotting with anti-HA and anti-GST antibodies. (**g**) Cln-CDK phosphorylates Rim15 *in vitro*. Cln2-HA_3_-CDK and Clb5-HA_3_-CDK immunoprecipitated from exponentially growing yeast cells were tested for their capacity to phosphorylate *in vitro* a bacterially purified GST-Rim15 fragment encompassing amino acids 944–1149 of Rim15 (Rim15^KI^, for *k*inase *i*nsert), GST alone or the GST-Sic1^1-100^ fragment (see Figure 2c). Sypro-Ruby staining of the gel (left panel) and the corresponding autoradiograph [^32^P] are shown. GST-Sic1^1-100^ served as positive control for both Clb5-/Cln2-CDK activities. Asterisks denote respective autophosphorylation bands. (**h**) Inactivation of Cdc28^as^ correlates with hypophosphorylation of Rim15-pThr^1075^ *in vivo*. Cells with the indicated genotypes were grown exponentially and then treated (+), or not (−), for 1 h with 1NM-PP1. The levels of Rim15-myc_13_ and the phosphorylation of the residue Thr^1075^ in Rim15 were determined via immunoblot analysis using anti-myc and phospho-specific anti-Rim15-pThr^1075^ antibodies, respectively. Cells expressing Rim15^T1075A^-myc_13_ were used as control.

To substantiate our model in which TORC1 regulates the binding between Cks1 and its docking site at Thr^173^ in Sic1 via Mpk1 and the Gwl pathway (Rim15-Igo1/2-PP2A^Cdc55^), we next quantified the relative levels of Sic1 that were precipitated with Cks1 in exponentially growing and rapamycin-treated WT, *mpk1*Δ and *cdc55*Δ cells. As expected, inhibition of TORC1 strongly stimulated the relative affinity between Sic1 and Cks1 (see also Figure 2e and f), and this depended on the presence of Mpk1 and the Thr^173^ residue in Sic1, while loss of Cdc55 strengthened the respective interaction (Figure 4a). Thus, by inhibiting Mpk1 and the Gwl pathway, TORC1 antagonizes Sic-Thr^173^ phosphorylation, and, as a consequence, the sequestration of Cks1 by Sic1 in exponentially growing cells. Nevertheless, exponentially growing cells also contain low levels of Sic1-pThr^173^ (see above) and the mutation of Thr^173^ in Sic1 *per se*, whether combined or not with *cdc55*Δ, measurably weakened the affinity between Sic1 and Cks1 in exponentially growing cells (Figure 4a). We considered it therefore possible that an additional mechanism exists, which fine-tunes the Sic1-Thr^173^ phosphorylation levels in proliferating cells. To analyze whether this presumed mechanism operates at different stages of cycling cells, we determined the levels of both Sic1 and Sic1-pThr^173^ in synchronized cells that were released from an α-factor arrest. Surprisingly, the phosphorylation of Sic1-Thr^173^ was clearly cell cycle regulated. It correlated closely with the relative amount of Sic1, being high in G_1_ cells and low in cells that have entered S-phase, as judged by the sequential waves of Cln2 and Clb5 in these cells (Figure 4b). Notably, in this context, both phosphorylation analyses of the Clb5-CDK target Sld2 and standard flow cytometric experiments previously revealed that, when released from an α-factor arrest, Sic1^T173A^-expressing cells enter S phase significantly earlier than WT cells (Figure 5b and e in Adrover *et al.* [30]). Combined with our finding that the Cln-/Clb5/6-CDK activity (assayed by monitoring the relative phosphorylation of Thr^5^ in Sic1/Sic1^T173A^) raises more rapidly and subsequently oscillates at a significantly higher level in *sic1*^*T173A*^ than in WT cells when released from an α-factor arrest (Supplementary Figure S4), these data indicate that the phosphorylation of Thr^173^ in Sic1 is also physiologically relevant in proliferating cells.

Interestingly, the rapid disappearance of phosphorylated Sic1-pThr^173^ species (when cells were released from the α-factor arrest) temporally coincided with a significant decrease in the level of the active version of the PP2A^Cdc55^-inhibitory endosulfine Igo1 (that is, Igo1-pSer^64^) [20]. Because the latter represents a direct readout for the activity of the Gwl Rim15 *in vivo* [31], this indicated that Rim15 may be inhibited by CDK at the onset of the G_1_–S transition phase and be released from this inhibition when CDK is low (that is, in late mitosis and G_1_). Consistent with this idea, inactivation of Cdc28^as^ with 1NM-PP1 stimulated the activity of Rim15, which was not due to indirect effects on TORC1, because the phosphorylation of Thr^737^ in Sch9, a proxy for TORC1 activity, remained largely constant throughout the experiment (Figure 4c). Notably, the activation of Rim15 following CDK inhibition was paralleled by an increased appearance of Rim15^KD^-GFP in the nuclei of exponentially growing cells (Figure 4d), which also fits well with the model that Rim15 activation requires its transfer into the nucleus [24]. Finally, in line with the implicit assumption that CDK inhibits Rim15 by preventing its accumulation in the nucleus, the dynamics of Rim15 inactivation correlated well with its relocation from the nucleus to the cytoplasm when cells were released from an α-factor arrest (Figure 4b and e).

The nucleocytoplasmic distribution of Rim15 is intimately controlled by the phosphorylation status of two residues (Ser^1061^ and Thr^1075^) within adjacent classical 14-3-3-binding domains that mediate cooperative association with dimeric 14-3-3 proteins in the cytoplasm [32]. Both residues are targeted by known protein kinases, namely, the TORC1 effector Sch9 (Rim15-Ser^1061^) and the phosphate-responsive cyclin-CDK Pho80-Pho85 module (Rim15-Thr^1075^) [21, 23, 24]. Because both CDKs, Pho85 and Cdc28, are known to redundantly phosphorylate the same CDK consensus sites in some of their effector proteins [33, 34], we speculated that Cdc28 could, in a most simple model, modulate the subcellular localization of Rim15 by regulating the phosphorylation of the previously identified Rim15-Thr^1075^ residue together with Pho85. Supporting this hypothesis, we found Rim15 to specifically interact with Cln2, but not with Clb5 or the control protein Lst7, when probed in exponentially growing cells (Figure 4f). Moreover, Cln2-Cdc28, but not Clb5-Cdc28, was able to phosphorylate Rim15 *in vitro* (Figure 4g), and inactivation of Cdc28^as^ with 1NM-PP1, similar to the loss of Pho85 [24], significantly reduced the Rim15-pThr^1075^ levels *in vivo* (Figure 4h). Therefore, Cln-CDK antagonizes Rim15, in parallel to or possibly downstream of Pho80–85 [35], by phosphorylation of its Thr^1075^ residue. Together with the TORC1/Sch9-mediated phosphorylation of Ser^1061^ in Rim15, this ensures cooperative cytoplasmic retention of inactive Rim15 by 14-3-3 proteins, which is also validated by the observation that Cln-CDK inactivation and rapamycin treatment triggered nuclear accumulation of Rim15^KD^ in an additive manner (Figure 4d). Taken together, our current study advocates a model in which the Gwl Rim15 coordinates the G_1_–S transition in response to cell cycle and nutritional cues by integrating the signals that emanate from Cln-CDK and TORC1-Sch9, respectively.

### Analogies between the roles of budding yeast and metazoan Gwl pathways

The budding yeast and metazoan Gwl pathways are organized in a strikingly similar way. Indeed, as shown here and in previous studies [36], they are both subjected to a direct control by CDKs, which likely serves to sharpen the onset of the Gwl-controlled cell cycle transition(s). Curiously, however, the main role of the Gwl pathway appears to be different in budding yeast and metazoans: yeast Gwl preferably antagonizes G_1_–S [17, 37, 38], while metazoan (and likely also fission yeast) Gwl primarily promotes the G_2_–M cell cycle transition [17, 39–41]. The Gwl pathway may therefore have been rewired during the evolution of higher eukaryotes to control G_2_–M instead of G_1_–S. Alternatively, the observed divergent roles of the Gwl pathways in budding yeast and metazoans could have evolved from an ancestral version of this pathway that functioned in both G_1_–S and G_2_–M, which raises the possibility that higher eukaryotes may have retained some aspects of the Gwl-mediated G_1_–S control mechanism identified in this study. In this context, it is compelling that mammalian cells express the cyclin-CDK2 inhibitor p27^Kip1^, which is structurally similar to Sic1 and also functions in G_0/1_–S cell cycle transition control [42]. Moreover, similar to Sic1, p27^Kip1^ is flagged for degradation by the combined action of cyclin-CDK, Cks1 and the SCF^Skp2^ ubiquitin ligase complex, while it is stably maintained in G_0_ cells only when phosphorylated at specific amino-acid residues [43]. Considering the importance of p27^Kip1^ and Cks1 in suppressing and promoting cancer progression, respectively [43, 44], these analogies warrant future studies that address a potentially conserved role of the Gwl pathway in G_1_–S transition control.

## Materials and Methods

### Strains, plasmids and growth conditions

Strains and plasmids are listed in Supplementary Tables S1 and S2, respectively. Notably, Sic1 alleles exhibit several polymorphisms, one of which includes an additional Glu residue at position 129 of the protein in some strain backgrounds, including the JK9-3D used here (see *Saccharomyces* Genome Database). To remain consistent with the previously published literature, we refer to Sic1-Thr^173^ throughout our study although the respective residue corresponds to Sic1-Thr^174^ in JK9-3D. *Saccharomyces cerevisiae* yeast cells were pregrown overnight at 30 °C in standard rich medium (YPD) with 2% glucose or in synthethic defined medium (0.17% yeast nitrogen base, 0.5% ammonium sulfate, 0.2% appropriate dropout mix (USBiological, Salem, MA, USA) and 2% glucose) for maintenance of plasmids. Prior to the experiments, cells were diluted to an OD_600_ of 0.15 and grown until they reached an OD_600_ of 0.6. Rapamycin (LC Laboratories, Woburn, MA, USA), 1NM-PP1 (Cayman Chemicals, Ann Arbor, MI, USA), and α-factor (KareBay Biochem, Monmouth Junction, NJ, USA) were used at final concentrations of 200 ng ml^−1^, 0.5 μM, and 5 μg ml^−1^, respectively.

### Immunoblot analyses

Protein extraction was performed by mild alkali treatment of cells followed by boiling in standard electrophoresis buffer [45]. Sodium dodecyl sulfate-polyacrylamide gel electrophoresis and immunoblot analyses were performed according to standard protocols. Phosphorylation of Sic1 variants was assessed by loading whole-cell extracts on 9% sodium dodecyl sulfate-polyacrylamide gel electrophoresis gels containing 50 μM Phos-tag (Wako, Neuss, Germany). The sources and dilutions of antibodies used are listed in Supplementary Table S3.

### Co-immunoprecipitation

For co-IP analyses, lysates were prepared by disruption of frozen cells in lysis buffer (50 mM TRIS (pH 7.5), 1 mM EDTA, 150 mM NaCl, 0.5% NP40 and 1× protease and phosphatase inhibitor cocktails (Roche, Rotkreuz, Switzerland)) with glass beads (0.5 mm diameter) using a Precellys cell disruptor (Bertin Technologies, Montigny-le-Bretonneux, France) and subsequent clarification by centrifugation (5 min at 14 000 r.p.m.; 4 °C). HA- and myc-tagged proteins were immunoprecipitated with an anti-HA and anti-myc magnetic matrix (Pierce, Lausanne, Switzerland), respectively. GST-tagged proteins were pulled down with glutathione-sepharose beads (GE Healthcare, Otelfingen, Switzerland).

### In vitro kinase assays

Protein kinases were immunoprecipitated as above. For CDK assays, Cln2-HA_3_ or Clb5-HA_3_ immunocomplexes were incubated at 30 °C for 20 min (or for the times indicated) in kinase buffer (125 mM TRIS pH 7.5, 50 mM MgCl_2_, 2.5 mM dithiothreitol and either 10 mM ATP for non-radioactive kinase assays or 20 μM ATP with 10 μCi of γ-^32^P ATP (Hartmann Analytic, Braunschweig, Germany) for radioactive kinase assays). The reactions were stopped with sample buffer and boiled for 5 min. For the CDK inhibition assays, the immunoprecipitated kinase complexes were incubated for 1 h on ice with GST-Sic1, GST-Sic1^T173A^ or GST-Sic1^R262A L264A^ (that were subjected to *in vitro* phosphorylation by Mpk1 before use) and then assayed in CDK assays using GST-Sic1^1-100^ as substrate. The levels of Thr^5^ in Sic1 were detected with phosphospecific anti-Sic1-pThr^5^ antibodies.

### Fluorescence microscopy

Images were captured with an inverted Spinning Disk Confocal Microscope (VisiScope CSU-W1, Puchheim, Germany) that was equipped with an Evolve 512 (Photometrics, Tucson, AZ, USA) EM-CCD camera and a 100× 1.3 NA oil immersion Nikon CFI series objective (Egg, Switzerland).
