# Supplementary material for: TORC1 coordinates the conversion of Sic1 from a target to an inhibitor of cyclin-CDK-Cks1
Source: Cell Discov. 2017 May 2;3:17012–. doi: 10.1038/celldisc.2017.12 (PMC5412858; doi:10.1038/celldisc.2017.12)
Supplement: Supplementary Information [file celldisc201712-s1.pdf]

Supplementary Information

TORC1 Coordinates the Conversion of Sic1 from a Target to an Inhibitor of Cyclin-CDK-Cks1

Marta Moreno-Torres, Malika Jaquenoud, Marie-Pierre Péli-Gulli, Raffaele Nicastro, and Claudio De Virgilio

INVENTORY OF SUPPLEMENTARY INFORMATION

Supplementary Figures

Figure S1 *In vivo* and *in vitro* specificity of the anti-Sic1-pThr^{5}/-pThr^{33} antibodies.

Figure S2 Sic1^{T173A}-expressing *cdc4-2^{ts}* cells are not defective in the clearance of Cln1, Cln2 or Clb5 when treated with rapamycin.

Figure S3 Cln2-HA_3 and Clb5-HA_3 interact with Cdc28 *in vivo*.

Figure S4 Proliferating, Sic1^{T173A}-expressing cells exhibit enhanced levels of Cln-/Clb-CDK activity.

Supplementary Tables

Table S1. Strains used in this study
Table S2. Plasmids used in this study
Table S3. Antibodies used in this study

Supplementary References

Supplementary Figures

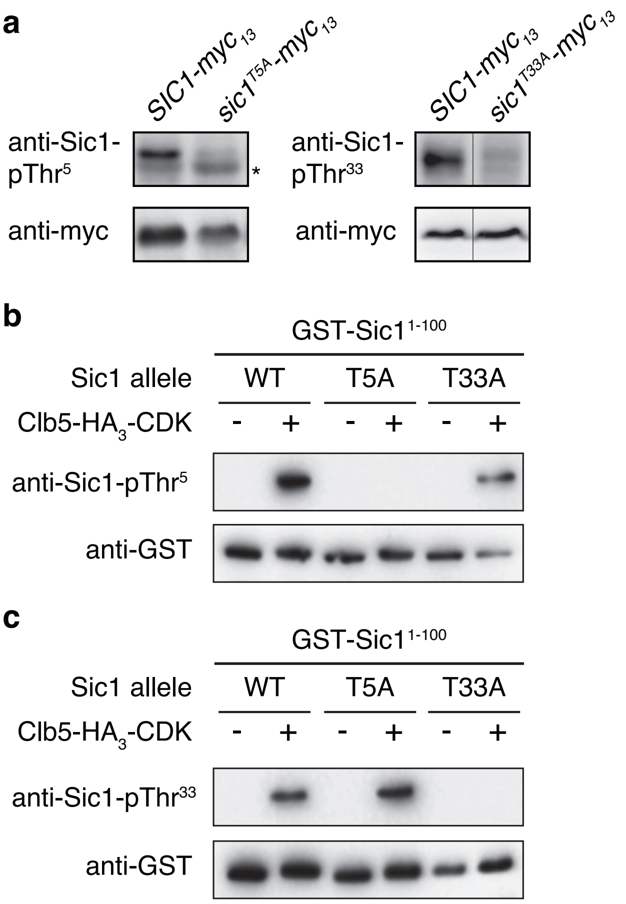

Figure S1 *In vivo* and *in vitro* specificity of the anti-Sic1-pThr^5/-pThr^{33} antibodies. (**a**) Sic1-pThr^5 and Sic1-pThr^{33} levels were determined by immunoblot analyses using respective phospho-specific antibodies and extract of exponentially growing cells that expressed plasmid-encoded versions of myc$_{13}$-tagged Sic1 and Sic1^{T5A} (left panels), or myc$_{13}$-tagged Sic1 and Sic1^{T33A} (right panels). The levels of the Sic1-myc$_{13}$ variants were determined by using polyclonal anti-myc antibodies. The asterisk denotes an unspecific band. (**b**, **c**) Clb5-HA$_3$-CDK immunocomplexes from exponentially growing yeast cells were used for *in vitro* kinase assays in which the bacterially-purified, N-terminal parts (encompassing the first 100 amino acids) of Sic1 (WT), Sic1^{T5A} (T5A), and Sic1^{T33A} (T33A) served as substrates. Sic1-pThr^5 (b) and Sic1-pThr^{33} (c) levels were determined by immunoblot analyses using respective phospho-specific antibodies and the indicated GST-Sic1$^{1\text{-}100}$ variants that have been phosphorylated (+), or not (-), by Clb5-HA$_3$-CDK. The input levels of the GST-Sic1$^{1\text{-}100}$ variants were determined by using polyclonal anti-GST antibodies.

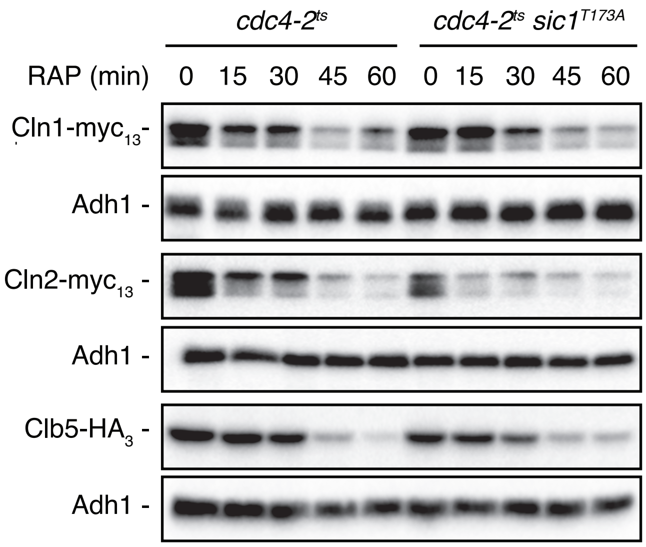

Figure S2 Sic1^{T173A}-expressing *cdc4-2^{ts}* cells are not defective in the clearance of Cln1, Cln2 or Clb5 when treated with rapamycin. *cdc4-2^{ts}* and *cdc4-2^{ts} sic1^{T173A}* strains expressing Cln1-myc$_{13}$, Cln2-myc$_{13}$, or Clb5-HA$_3$ were grown as in Fig. 1A. the levels of the tagged proteins were determined by immunoblot analyses using monoclonal anti-myc or anti-HA antibodies. Adh1 levels served as loading control.

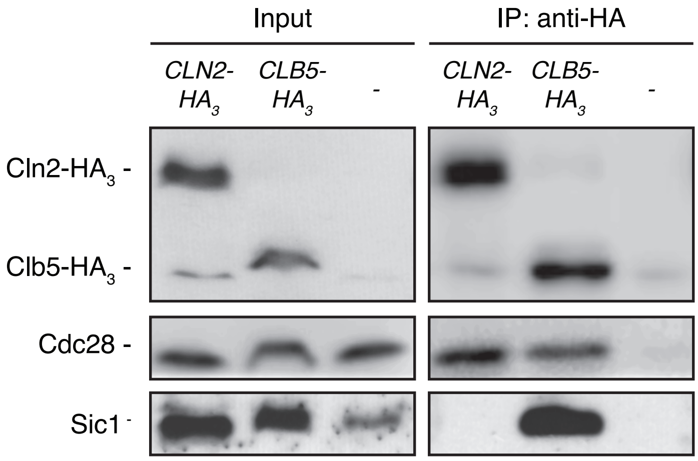

Figure S3 Cln2-HA$_3$ and Clb5-HA$_3$ interact with Cdc28 *in vivo*. Plasmid-expressed, HA$_3$-tagged Cln2 or Clb5 were immunoprecipitated (IPed) from extracts of exponentially growing wild-type cells. Cell lysates (Input) and anti-HA immunoprecipitates (IP: anti-HA) were analyzed by immunoblotting with anti-HA (top panels), anti-Cdc28 (panels in the middle), and anti-Sic1 (bottom panels) antibodies. Please note that both Cln2-HA$_3$ and Clb5-HA$_3$ interact with Cdc28, while only Clb5-HA$_3$ is able to bind Sic1 as expected. Neither Cdc28 nor Sic1 were recovered in anti-HA immunoprecipitates from extracts of cells that carried an empty plasmid (-; 3rd lanes in all panels).

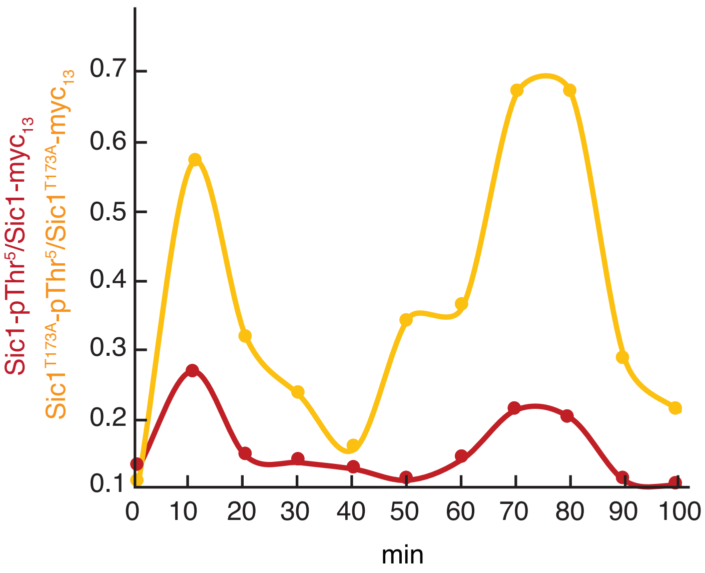

Figure S4 Proliferating, Sic1^{T173A}-expressing cells exhibit enhanced levels of Cln-/Clb5/6-CDK activity. For synchronization, exponentially growing WT cells expressing genomically-tagged Sic1-myc$_{13}$ or Sic1^{T173A}-myc$_{13}$ were treated for 2 h with α-factor (5 µg ml^{-1}). Following α-factor release, samples were collected at the indicated time points. The phosphorylation levels of Thr^5 in Sic1 and Sic1^{T173A} were determined by using phosphospecific anti-Sic1-pThr^5 antibodies and normalized with respect to the total levels (quantified by using anti-myc antibodies) of Sic1-myc$_{13}$ and Sic1^{T173A}-myc$_{13}$, respectively.

Supplementary Tables

Table S1. Strains used in this study

Strain	Genotype	Source	Figure
JK9-3D	*MAT***a**, *leu2, his4, trp1, ura3, rme1, GAL, HMLa*	Ref. (1)	2A/B, 3B, 2C, 4G, S1B/C, S3
RL343-E1	[JK9-3D] *his3*, *HIS4*, *cdc28*Δ, pRS416-*cdc28*^{as} (F88G)	Ref. (2)	4C
YMM114	[JK9-3D] *cdc4-2*^{ts}*::kanMX*	Ref. (3)	1A/C
YMM118-2D	[JK9-3D] *cdc4-2*^{ts}*::kanMX, sic1*^{T173A}*, EMP46::natMX*	Ref. (3)	1A/C
YMM67-1C	[JK9-3D] *sic1*Δ*::kanMX*	Ref. (3)	1A/B, S1A
YMM237-1A	[JK9-3D] *cdc4-2*^{ts}*::kanMX, cdc28*Δ*::kanMX,* pRS416-*cdc28*^{as}	This study	1B/D
YMM250-10C	[JK9-3D] *cdc4-2*^{ts}*::kanMX, cdc28*Δ*::kanMX,* pRS416-*cdc28*^{as}*, sic1*^{T173A}*, EMP46::natMX*	This study	1B/D
YMM246-1C	[JK9-3D] *cdc4-2*^{ts}*::kanMX, CLB5-HA*$_{3}$*::TRP1, CLN1-myc*$_{13}$*::kanMX*	This study	S2
YMM247-4B	[JK9-3D] *cdc4-2*^{ts}*::kanMX, CLB5-HA*$_{3}$*::TRP1, CLN1-myc*$_{13}$*::kanMX, sic1*^{T173A}*, EMP46::natMX*	This study	S2
YMM249-2B	[JK9-3D] *cdc4-2*^{ts}*::kanMX, CLB5-HA*$_{3}$*::TRP1, CLN2-myc*$_{13}$*::kanMX*	This study	S2
YMM252-2A	[JK9-3D] *cdc4-2*^{ts}*::kanMX, CLB5-HA*$_{3}$*::TRP1, CLN2-myc*$_{13}$*::kanMX, sic1*^{T173A}*, EMP46::natMX*	This study	S2
MJA4090	[JK9-3D] *cdc4-2*^{ts}*::kanMX, CLB5-HA*$_{3}$*::TRP1*	This study	S2
MJA4091	[JK9-3D] *cdc4-2*^{ts}*::kanMX, CLB5-HA*$_{3}$*::TRP1, sic1*^{T173A}*, EMP46::natMX*	This study	S2
YMM91	[JK9-3D] *sic1*^{T173A}*, EMP46::natMX*	Ref. (3)	2A/B, 3B,
MJA490	[JK9-3D] *CKS1-HA*$_{3}$*::kanMX*	This study	2D-F, 4A
MJA491	[JK9-3D] *CKS1-HA*$_{3}$*::kanMX, sic1*^{T173A}*, EMP46::natMX*	This study	2D-E, 4A
MJA524-2B	[JK9-3D] *sic1*$^{R262A/L264A}$*-myc*$_{13}$*::kanMX*	This study	3A
YMM63	[JK9-3D] *SIC1-myc*$_{13}$*::kanMX*	Ref. (3)	3A, 4B, S4
YMM98	[JK9-3D] *sic1*^{T173A}*-myc*$_{13}$*::kanMX, EMP46::natMX*	Ref. (3)	3A, S4
MJA523	[JK9-3D] *sic1*$^{R262A/L264A}$*, EMP46::natMX*	This study	3B
MJA536	[JK9-3D] *cln1Δ::kanMX, cln2Δ::kanMX*	This study	3B
MJA528	[JK9-3D] *sic1*$^{T173A/R262A/L264A}$*, EMP46::natMX*	This study	3B
YMM232-6A	[JK9-3D] *clb5Δ::kanMX, clb6Δ::kanMX, sic1*^{T173A}*, EMP46::natMX*	This study	3B
MJA544-2B	[JK9-3D] c*ln1Δ::kanMX, cln2Δ::kanMX, sic1*^{T173A}*, EMP46::natMX*	This study	3B
YMM253-11A	[JK9-3D] *clb5Δ::kanMX, clb6Δ::kanMX*	This study	3B
MJA547	[JK9-3D] *clb5Δ::kanMX, cln1Δ::kanMX, cln2Δ::kanMX, sic1*^{T173A}*, EMP46::natMX*	This study	3B
MJA545	[JK9-3D] *CKS1-HA*$_{3}$*::kanMX, CLB5-myc*$_{13}$*::kanMX, sic1*^{T173A}*, EMP46::natMX*	This study	3D
MJA546	[JK9-3D] *CKS1-HA*$_{3}$*::kanMX, CLB5-myc*$_{13}$*::kanMX*	This study	3D
MJA531	[JK9-3D] *CKS1-HA*$_{3}$*::kanMX, CLN2-myc*$_{13}$*::kanMX, sic1*^{T173A}*, EMP46::natMX*	This study	3E
MJA530	[JK9-3D] *CKS1-HA*$_{3}$*::kanMX, CLN2-myc*$_{13}$*::kanMX,*	This study	3E
YMM231-1A	[JK9-3D] *CKS1-HA*$_{3}$*::kanMX, mpk1Δ::kanMX*	This study	4A
YMM230-8A	[JK9-3D] *CKS1-HA*$_{3}$*::kanMX, cdc55Δ::natMX*	This study	4A
YMM233-3A	[JK9-3D] *CKS1-HA*$_{3}$*::kanMX, cdc55Δ::natMX, sic1*^{T173A}*, EMP46::natMX*	This study	4A
MJA518	[JK9-3D] *cdc28*Δ*, pRS416-cdc28*^{as}	This study	4C
MJA519	[JK9-3D] *cdc28*Δ*, pRS416-cdc28*^{as}*, rim15Δ::kanMX*	This study	4C/H
YMM58-1B	[JK9-3D] *rim15Δ::kanMX*	This study	4E/F
MM3D	[JK9-3D] *cdc28*Δ, pRS416-*cdc28*^{as}*, rim15Δ::kanMX, LEU2::CYC1p-HHF2-tDimer*	This study	4D

Table S2. Plasmids used in this study

Plasmid	Genotype	Source	Figure
pRS415	CEN, *LEU2*	Ref. (5)	
pMJA2881	[pRS415] *ADH1p-SIC1-myc*$_{13}$	This study	S1A
pMJA3173	[pRS415] *ADH1p-sic1*^{T5A}*-myc*$_{13}$	This study	S1A
pMJA3174	[pRS415] *ADH1p-sic1*^{T33A}*-myc*$_{13}$	This study	S1A
pMJA2995	[pRS415] *ADH1p-SIC1*$^{150\text{-}285}$*-myc*$_{13}$	This study	2F
pMJA2996	[pRS415] *ADH1p-sic1*$^{150\text{-}285\text{ - }T173A}$*-myc*$_{13}$	This study	2F
pRS416	CEN, *URA3*	Ref. (5)	S3
pMJA3038	[pRS416] *ADH1p-CLB5-HA*$_{3}$	This study	2B/C, 3A, 4F/G, S3, S1B/C, S3
YCplac33	*CEN, URA3*	Ref. (6)	
JCE456	[YCplac33] *ADH1p-CLN2-HA*$_{3}$	Ref. (7)	2A/C, 4D, 4F/G, S3
pAS2654	[YCplac33] *ADH1p-LST7-HA*$_{3}$	Ref. (8)	4F
pGEX	*GST*	Ref. (9)	4G, S1B/C
pMMT2629	[pGEX] *GST-SIC1*	This study	2C
pMMT2630	[pGEX] *GST-sic1*^{T173A}	This study	2C
pMJA3029	[pGEX] *GST-sic1*$^{R262A/L264A}$	This study	2C
pMJA3037	[pGEX] *GST-SIC1*$^{1\text{-}100}$	This study	2C, 3A, 4G, S1B/C
pMJA3219	[pGEX] *GST-sic1*$^{T5A(1\text{-}100)}$	This study	S1B/C
pMJA3220	[pGEX] *GST-sic1*$^{T33A(1\text{-}100)}$	This study	S1B/C
pVW995	[pGEX] *GST-RIM15*$^{944\text{-}1149}$	Ref. (10)	4G
pVW827	CEN, *LEU2, ADH1p-GST-RIM15*	Ref. (10)	4F
pVW904	2μ, *LEU2, TDH3p-RIM15-myc*$_{13}$	Ref. (10)	4H
pVW910	2μ, *LEU2, TDH3p-rim15* ^{T1075A}*-myc*$_{13}$	Ref. (10)	4H
pFD1008	CEN, *TRP1, ADH1p-rim15*^{K823Y}*-GFP*	Ref. (10)	4D/E

Table S3. Antibodies used in this study

Name	Dilution	Source
Anti-Sic1	1:1’000	sc-50441 Santa Cruz
Anti-Adh1	1:200’000	Calbiochem
Anti-Sic1-pThr^{173}	1:1’000	GenScript
Anti-Sic1-pThr^{5}	1:1’000	GenScript
Anti-Sic1-pThr^{33}	1:1’000	GenScript
Anti-myc	1:3’000	9E10; sc-40; Santa Cruz
Anti-HA	1:1’000	Enzo Life Sciences
Anti-GST	1:3’000	Bethyl Laboratories
Anti-Igo1-pSer^{64}	1:1’000	GenScript
Anti-Igo1	1:1’000	Eurogentec
Anti-Cln2	1:1’000	Santa Cruz
Anti-Cdc28	1:300	Santa Cruz
Anti-Clb5	1:1’000	Santa Cruz
Anti-Sch9-pThr^{737}	1:1’0000	GenScript
Anti-Sch9	1:1’000	GenScript
Anti-Rim15-pThr^{1075}	1:10’000	Eurogentec
Goat anti-rabbit IgG HRP	1:3’000	Biorad
Goat anti-mouse HRP	1:3’000	Biorad
Donkey anti-goat HRP	1:5’000	Abcam
Goat anti-mouse IgG-Fcy HRP	1:5’000	Jackson Immunoresearch
Goat anti-mouse IgG, light chain HRP	1:5’000	Jackson Immunoresearch

Supplementary References

1. Heitman J, Movva NR, & Hall MN (1991) Targets for cell cycle arrest by the immunosuppressant rapamycin in yeast. *Science* 253(5022):905-909.
2. Bodenmiller B*, et al.* (2010) Phosphoproteomic analysis reveals interconnected system-wide responses to perturbations of kinases and phosphatases in yeast. *Sci. Signal.* 3(153):rs4.
3. Moreno-Torres M, Jaquenoud M, & De Virgilio C (2015) TORC1 controls G_1-S cell cycle transition in yeast via Mpk1 and the greatwall kinase pathway. *Nat. Commun.* 6:8256.
4. Pedruzzi I*, et al.* (2003) TOR and PKA signaling pathways converge on the protein kinase Rim15 to control entry into G_0. *Mol. Cell* 12(6):1607-1613.
5. Brachmann CB*, et al.* (1998) Designer deletion strains derived from *Saccharomyces cerevisiae* S288C: a useful set of strains and plasmids for PCR-mediated gene disruption and other applications. *Yeast* 14(2):115-132.
6. Gietz RD & Sugino A (1988) New yeast-*Escherichia coli* shuttle vectors constructed with *in vitro* mutagenized yeast genes lacking six-base pair restriction sites. *Gene* 74(2):527-534.
7. Quilis I & Igual JC (2012) Molecular basis of the functional distinction between Cln1 and Cln2 cyclins. *Cell Cycle* 11(16):3117-3131.
8. Péli-Gulli MP, Sardu A, Panchaud N, Raucci S, & De Virgilio C (2015) Amino Acids Stimulate TORC1 through Lst4-Lst7, a GTPase-Activating Protein Complex for the Rag Family GTPase Gtr2. *Cell Rep* 13(1):1-7.
9. Smith DB & Johnson KS (1988) Single-step purification of polypeptides expressed in *Escherichia coli* as fusions with glutathione S-transferase. *Gene* 67(1):31-40.
10. Wanke V, Pedruzzi I, Cameroni E, Dubouloz F, & De Virgilio C (2005) Regulation of G_0 entry by the Pho80-Pho85 cyclin-CDK complex. *EMBO J.* 24(24):4271-4278.
